# Metal‐Driven Autoantifriction Function of Artificial Hip Joint

**DOI:** 10.1002/advs.202301095

**Published:** 2023-07-06

**Authors:** Qiaoyuan Deng, Qingguo Feng, Peipei Jing, Donglin Ma, Mengting Li, Yanli Gong, Yantao Li, Feng Wen, Yongxiang Leng

**Affiliations:** ^1^ Institute of Biomedical Engineering Key Laboratory of Advanced Technologies of Materials Ministry of Education College of Medicine Southwest Jiaotong University Chengdu Sichuan 610031 China; ^2^ Key Laboratory of Advanced Material of Tropical Island Resources of Educational Ministry School of Materials Science and Engineering Hainan University Haikou Hainan 570228 China; ^3^ College of Physics and Engineering Chengdu Normal University Chengdu Sichuan 611130 China; ^4^ Hainan Provincial Fine Chemical Engineering Research Center, School of Chemical Engineering and Technology Hainan University Haikou Hainan 570228 P. R. China

**Keywords:** artificial hip joints, autoantifriction, graphite‐like carbon films, TiN*_X_*–Cu films, wear resistance

## Abstract

The service life of an artificial hip joint is limited to 10–15 years, which is not ideal for young patients. To extend the lifespan of these prostheses, the coefficient of friction and wear resistance of metallic femoral heads must be improved. In this study, a Cu‐doped titanium nitride (TiN*_X_*–Cu) film with “autoantifriction” properties is deposited on a CoCrMo alloy via magnetron sputtering. When delivered in a protein‐containing lubricating medium, the Cu in TiN*_X_*–Cu quickly and consistently binds to the protein molecules in the microenvironment, resulting in the formation of a stable protein layer. The proteins adsorbed on the TiN*_X_*–Cu surface decompose into hydrocarbon fragments owing to the shear stress between the Al_2_O_3_/TiN*_X_*–Cu tribopair. The synergistic effect of the catalysis of Cu and shear stress between the Al_2_O_3_/TiN*_X_*–Cu tribopair transforms these fragments into graphite‐like carbon tribofilms with an antifriction property. These tribofilms can simultaneously reduce the friction coefficient of the Al_2_O_3_/TiN*_X_*–Cu tribopair and enhance the wear resistance of the TiN*_X_*–Cu film. Based on these findings, it is believed that the autoantifriction film can drive the generation of antifriction tribofilms for lubricating and increasing the wear resistance of prosthetic devices, thereby prolonging their lifespan.

## Graphical abstract

The autoantifriction film can increase the wear resistance of the artificial hip joint. The Cu in autoantifriction film quickly binds to the protein molecules in the microenvironment, resulting in the formation of a protein layer. The combined effects of Cu catalysis and shear forces between the tribopair convert the proteins into a graphite‐like carbon film to lubricate the tribopair.

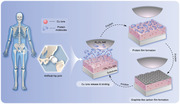

## 1 Introduction

Wear and corrosion have been major problems in the development of metallic materials for load‐bearing artificial joints, notably for metal‐on‐metal hip joints.^[^ ^1^ ^]^ To boost wear resistance and prevent the leaching of hazardous metal ions, typical hard and chemically inert films have been deposited on prosthetic surfaces.^[^ ^2^ ^]^ In the 1980s, titanium nitride (TiN) coatings were proposed as candidates to increase the wear resistance of artificial joints.^[^ ^3^ ^]^ TiN films enhance the mechanical properties,^[^ ^4^ ^]^ corrosion resistance,^[^ ^5^ ^]^ and biocompatibility^[^ ^4^, ^6^ ^]^ of artificial hip joints. TiN‐coated prosthetic femoral heads have been successfully used in clinical applications.^[^ ^7^ ^]^ However, retrieval analyses have indicated that after several years of implantation, TiN‐coated prosthetic femoral heads fail owing to partial film delamination.^[^ ^8^ ^]^ All retrieved prosthetic femoral heads with TiN coatings exhibit microdefects such as pinholes or small metal droplets.^[^ ^9^ ^]^ Hauert^[^ ^10^ ^]^ proposed that their delamination arose from stress corrosion cracking (SCC), which is caused by the interlayer corrosion of the substrate/coating induced by the physiological medium diffusing through the pinholes in the coatings. Currently, enhancing the compactness of the film (reducing pinholes) and producing multilayer films are the two most effective methods for preventing coating delamination.^[^ ^11^ ^]^ In addition, the three‐body abrasion caused by TiN debris delaminated from the surface of the coating reduces the wear resistance and service life of the coating in the clinic.^[^ ^9^, ^11^ ^]^ Therefore, we envisioned that improving the lubricating qualities of the tribointerface of the joint and boosting the wear resistance of the TiN coatings would be an effective way to extend the life of the TiN‐coated joint.

Shellfish such as shrimps and crabs can absorb mineral ions (such as Ca^2+^ and Mg^2+^) from their surroundings and naturally develop a protective, hard shell after shelling. Theoretically, a similar method could be used to form a protective film on an artificial hip joint, as the microenvironment contains proteins when the joint is installed under physiological conditions. If the surface of the metal femoral head can adsorb proteins from the microenvironment and catalyze the formation of an antifriction layer that protects the prosthesis from wear, the service life of the artificial hip joint would be extended. Therefore, we propose an autoantifriction film with catalytic ability that uses proteins from the microenvironment to build a graphite‐like carbon film, which acts naturally as a lubricant layer to reduce mechanical wear.

**Figure 1:**
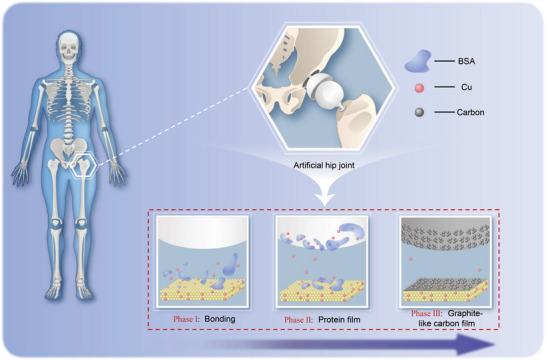
Schematic of the three stages of graphite‐like carbon tribofilm generation.

In this study, an autoantifriction TiN*_X_*–Cu film was deposited on the surface of a CoCrMo alloy by combining TiN and Cu (a metal catalyst). **Figure** **1** shows a schematic of the three‐stage generation of a graphite‐like carbon tribofilm. As the TiN*_X_*–Cu film is deposited on a prosthesis and administered to patients, copper ions are released from the film, and the proteins covalently bind to the surface of the tribopairs (phases I and II). Subsequently, the combined effects of copper catalysis and the shear forces between the tribopairs drive the proteins to transform into a graphite‐like carbon film (phase III). After these three stages, the auto‐generated carbon tribofilm with anti‐friction properties acts as a shield to lubricate the tribopairs and increase their wear resistance. Therefore, we believe that the autoantifriction ability of the TiN*_X_*–Cu film plays a crucial role in inducing self‐lubrication in vivo.

## 2 Results

### 2.1 Microstructure of TiN*_X_*–Cu Film

**Figure 2:**
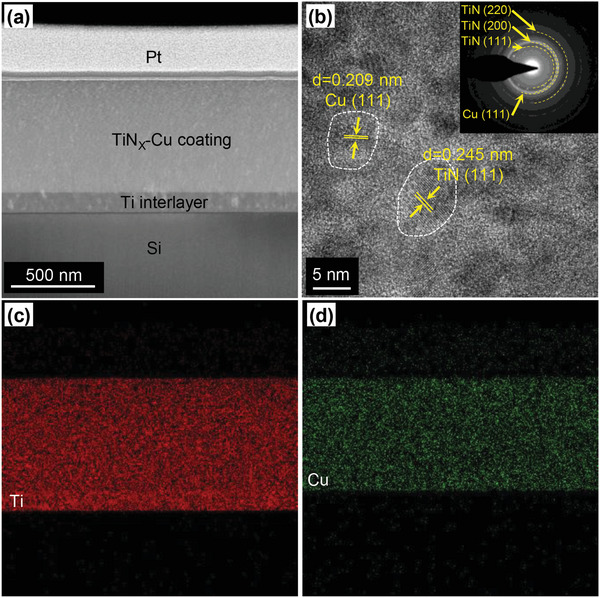
Structure of the TiN*_X_*–Cu film. a) Cross‐sectional image of the TiN*_X_*–Cu film prepared using a focused ion beam (FIB). The thin, bright layer with some dark areas above the silicon is a titanium transition layer between the film and the (150 nm thick) Si substrate. b) HRTEM image of the TiN*_X_*–Cu film, which was nanocrystalline with crystal sizes of <10 nm. c) Ti in the TiN*_X_*–Cu film. d) Cu distributed evenly in the TiN*_X_*–Cu film.

As shown in **Figure** **2**, the structure of the TiN*_X_*–Cu film was investigated using transmission electron microscopy (TEM). The cross section of the TiN*_X_*–Cu film (Figure 2a) exhibited a coating thickness of ≈800 nm. The high‐resolution transmission electron microscopy (HRTEM) image (Figure 2b) reveals a nanograin structure with a diameter of less than 10 nm. Cross‐sectional energy dispersion spectroscopy (EDS) of the TiN*_X_*–Cu film (Figure 2c,d) confirms the presence of Cu in the TiN layer. The surface morphology and Cu content (11.2 at%) of the TiN*_X_*–Cu film are shown in Figure S1, Supporting Information. The surface roughness, *R*_q_ (root mean square), of the films prepared in this study was ≈3 nm, as shown in Figure S9, Supporting Information.

### 2.2 Wear Resistance of the TiN and TiN*_X_*–Cu Films

**Figure 3:**
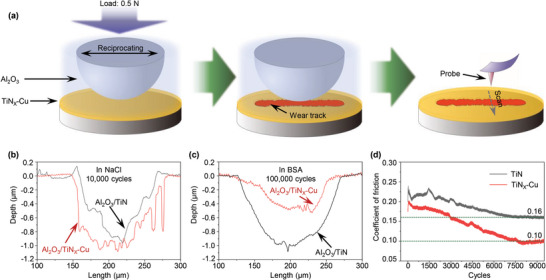
Wear resistance of the TiN and TiN*_X_*–Cu films coated on CoCrMo alloy disks in BSA solution and saline. a) Ball‐on‐disk wear‐testing schematic of TiN and TiN*_X_*–Cu films in BSA solution and saline. The profile of the wear track was characterized using a profilometer. b) Profile of the wear track on the TiN and TiN*_X_*–Cu films after wear testing in saline. c) Profile of the wear track on the TiN and TiN*_X_*–Cu films after wear testing in BSA solution. d) Friction coefficients of the Al_2_O_3_/TiN and Al_2_O_3_/TiN*_X_*–Cu tribopairs in BSA solution.

The wear test and corresponding profile are shown in **Figure** **3**a. A ball‐on‐disk tribometer was used to test the friction and wear of the TiN and TiN*_X_*–Cu films. Figure 3b shows the profile of the TiN and TiN*_X_*–Cu wear tracks, which have depths of ≈1 µm. The optical images of the wear track in the TiN and TiN*_X_*–Cu films shown in Figure S2a,b, Supporting Information, indicate that after 10 000 cycles of friction testing in saline solution, the TiN and TiN*_X_*–Cu films delaminated from the substrates. By contrast, even after 100 000 cycles of wear testing in bull serum albumin (BSA) solution, no films delaminated from the substrates (Figure S2c,d, Supporting Information). As shown in Figure 3c, the depth of the TiN*_X_*–Cu wear track was less than that of the Al_2_O_3_/TiN tribopairs in the BSA solution. Figure S3, Supporting Information, shows the wear indices of the TiN and TiN*_X_*–Cu films subjected to wear in saline and BSA solutions for 10 000 and 100 000 cycles, respectively. The wear index of the TiN*_X_*–Cu films was lower than that of the TiN films in BSA solutions. This indicates that the TiN*_X_*–Cu film exhibited higher wear resistance in BSA solution than the TiN film. According to the coefficient of friction (CoF) results shown in Figure 3d, the Al_2_O_3_/TiN*_X_*–Cu tribopair in the BSA solution stabilized at 0.1, while the Al_2_O_3_/TiN tribopair stabilized at 0.16. In addition, wear scars on the Al_2_O_3_ ball and wear tracks on the TiN*_X_*–Cu and TiN films were observed using optical microscopy (Figures S4 and S5, Supporting Information). Together, these results demonstrate that BSA protein molecules may interact with Cu during sliding and alter the friction interface of the Al_2_O_3_/TiN*_X_*–Cu tribopair, resulting in TiN*_X_*–Cu films with a higher wear resistance than the TiN films in the BSA solution. When sliding occurs between the tribopairs, a tribofilm emerges at the tribointerface to reduce the wear of the tribopair.^[^ ^12^ ^]^ For the Al_2_O_3_/TiN*_X_*–Cu tribopairs, some black debris was observed on the wear scar of the Al_2_O_3_ ball (Figure S4, Supporting Information); however, the tribofilm was too thin to observe using optical microscopy.

### 2.3 Characterization of the Graphite‐Like Carbon Tribofilm

**Figure 4:**
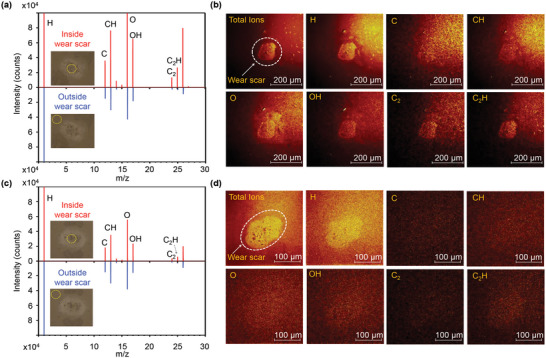
ToF‐SIMS spectra and elemental maps of the wear scar. a) ToF‐SIMS spectra of the interior and exterior of the wear scar on the Al_2_O_3_/TiN*_X_*–Cu tribopair. b) Mapping of carbonaceous debris on the interior and exterior of the wear scar on the Al_2_O_3_/TiN*_X_*–Cu tribopair. c) ToF‐SIMS spectra of the carbonaceous debris inside and outside the wear scar on the Al_2_O_3_/TiN*_X_*–Cu tribopair. d) No notable difference is apparent between the interior and exterior of the wear scars on the Al_2_O_3_/TiN tribopair.

Time‐of‐flight secondary‐ion mass spectrometry (ToF‐SIMS), a sensitive method for identifying the composition of surfaces, was used to validate the existence of a tribofilm on the tribointerface. **Figure** **4** shows the ToF‐SIMS spectra and elemental maps of the interior and exterior of the wear scars on the Al_2_O_3_/TiN*_X_*–Cu and Al_2_O_3_/TiN tribopairs. As shown in Figure 4a, the peaks in the ToF‐SIMS spectrum of the carbon‐containing debris (such as C, CH, C_2_, and C_2_H) inside the Al_2_O_3_ wear scar are significantly higher than those in the spectrum of the exterior of the wear scar. The 2D ToF‐SIMS image of the Al_2_O_3_/TiN*_X_*–Cu tribopair (Figure 4b) reveals that the debris mapping inside the wear scar was brighter than that of the exterior. The brightness of the debris mapping was positively correlated with the debris content. The results shown in Figure 4b indicate that, after the wear tests in BSA solution, the friction product formed on the wear scar of the Al_2_O_3_/TiN*_X_*–Cu tribopair contained more carbonaceous debris than in the surrounding areas. This demonstrates that the tribofilm formed on the wear scar of the Al_2_O_3_/TiN*_X_*–Cu tribopair after the wear tests in BSA solution was composed of carbon and hydrocarbon debris.

After wear testing in BSA solution, the intensity of the ToF‐SIMS spectra of the carbon and hydrocarbon debris inside the wear scar of the Al_2_O_3_ ball was similar to that of the debris outside the wear scar on the Al_2_O_3_/TiN tribopair (Figure 4c). Moreover, the brightness of the debris mapping of the inner wear scar is comparable to that of the outer wear scar of the Al_2_O_3_/TiN tribopair (Figure 4d). This suggests that after wear testing in BSA solution, it is more difficult for the Al_2_O_3_/TiN tribopair to accumulate carbon and hydrocarbon debris on the wear scar than for the Al_2_O_3_/TiN*_X_*–Cu tribopair. In general, the ToF‐SIMS results suggest that a tribofilm composed of carbon and hydrocarbon debris can form on the wear scars of the Al_2_O_3_/TiN*_X_*–Cu tribopair.

**Figure 5:**
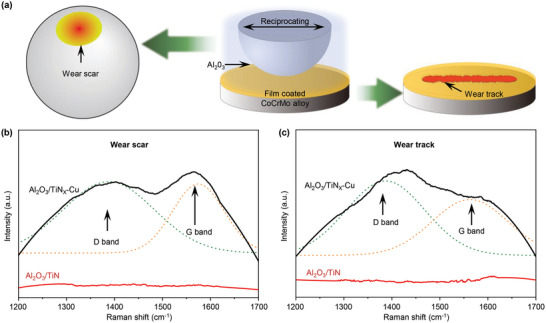
Raman spectra of the tribolayers formed after performing friction tests on Al_2_O_3_/TiN and Al_2_O_3_/TiN*_X_*–Cu in BSA solution. a) Wear scar on the Al_2_O_3_ ball and wear track on the films after ball‐on‐disk wear testing in BSA solution. b) Raman spectra of the debris found in the wear scar on the Al_2_O_3_ ball. c) Raman spectra of the wear track on the TiN and TiN*_X_*–Cu films.

Micro‐Raman spectra of the tribointerface (wear scar on Al_2_O_3_ and wear track on the TiN*_X_*–Cu and TiN films) were acquired to investigate the structure of the tribofilm, as shown in **Figure** **5**. For the Al_2_O_3_/TiN*_X_*–Cu tribointerface in BSA solution, the Raman spectra of the wear scar on Al_2_O_3_ contain a D band at ≈1380 cm^−1^ and a G band at ≈1580 cm^−1^ (Figure 5b). The TiN*_X_*–Cu wear track gave rise to a similar Raman spectrum, as shown in Figure 5c. The spectra indicate that the tribofilm generated at the interface of the Al_2_O_3_/TiN*_X_*–Cu tribopair is composed of carbon atoms with sp^2^‐bonds, also known as graphite‐like carbon. By contrast, the Raman spectra of the wear scar on Al_2_O_3_ (Figure 5b) and wear track on TiN (Figure 5c) on the tribointerface of the Al_2_O_3_/TiN tribopair exhibited no Raman activity in the BSA solution, indicating that no graphite‐like carbon was formed on the interface of the Al_2_O_3_/TiN tribopair after wear testing in the BSA solution.

**Figure 6:**
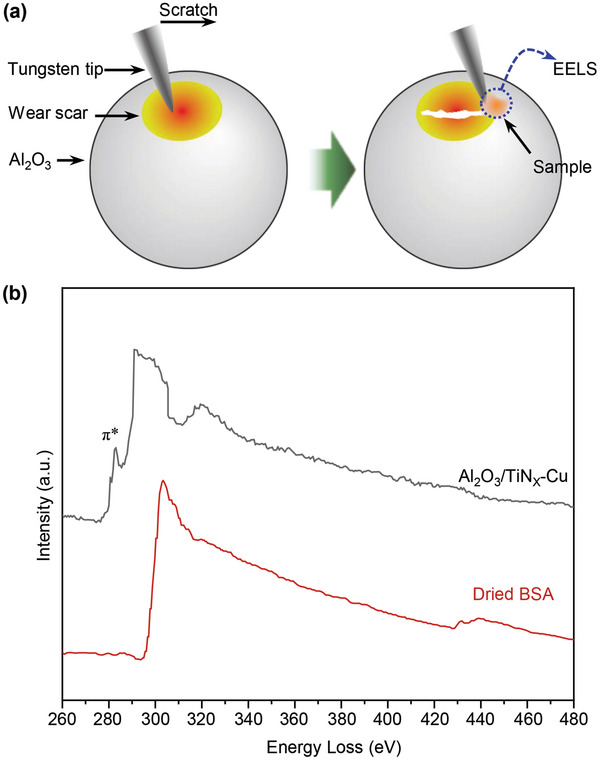
Schematic of TEM sample preparation and EELS results of the debris collected from the wear scar. a) Preparation schematic of the TEM sample used in this work. b) EELS spectrum of the sample collected from the Al_2_O_3_/TiN*_X_*–Cu tribopair and dried BSA, which reveals that the friction product generated on the friction interface of the Al_2_O_3_/TiN*_X_*–Cu tribopair is primarily composed of graphite‐like carbon.

The Raman spectra revealed that graphite‐like carbon developed on the Al_2_O_3_/TiN*_X_*–Cu tribointerface in BSA solution. However, under the same wear and friction conditions as those of the Al_2_O_3_/TiN*_X_*–Cu tribopair, the Al_2_O_3_/TiN tribointerface did not undergo carbon‐film formation. This suggests that graphite‐like carbon may not appear on the Al_2_O_3_/TiN tribointerface in BSA solution or that the amount of graphite‐like carbon generated is too low to be detected. To further confirm that the tribofilm of the Al_2_O_3_/TiN*_X_*–Cu tribopair consists of graphite‐like carbon, electron energy‐loss spectroscopy (EELS) was used to investigate the formation of the tribofilm. The sample preparation and test results are shown in **Figure** **6**. Briefly, a tungsten tip was used to scratch the wear scar on Al_2_O_3_ to transfer the wear debris from the wear product to the Cu grid, which served as the EELS sample (Figure 6a).

The π^⁎^ peak in the EELS spectrum at ≈285 eV is a characteristic signal of the graphitic structure.^[^ ^12^ ^]^ However, the π^⁎^ peak is absent in the EELS spectrum of the dried BSA (Figure 6b), indicating that the BSA molecule does not contain any six‐membered carbon rings (or the content is too low to be detected). Furthermore, the energy of the electrons used during EELS testing (such as the voltage and electron‐beam density) does not severely damage or carbonize the protein structure. The π^⁎^ peak at ≈285 eV in the spectrum of the Al_2_O_3_/TiN*_X_*–Cu tribopair indicates that the structure of the tribofilm on the tribointerface consists of carbonaceous material with sp^2^ character. This finding is consistent with the Raman results, as the G band corresponds to graphite‐like characteristics (Figure 5b). This illustrates that the shear force between the Al_2_O_3_/TiN*_X_*–Cu tribopair can break the structure of the BSA molecules and generate graphite‐like carbon on the surface of the tribointerface. The EELS and Raman results confirmed that the TiN*_X_*–Cu film could convert BSA molecules into a carbonaceous tribofilm at the tribointerface. Owing to its sp^2^ character, the carbonaceous film generated in this study is referred to as a “graphite‐like carbon film,” which reduces the CoF between tribopairs (Figure 3d) and eliminates the wear of the tribopairs (Figure 3c).

### 2.4 Simulation Studies of the Molecules on the TiN*_X_*–Cu Film

Density functional theory (DFT) and ab initio molecular dynamics (AIMD) simulations using the Vienna ab initio simulation package (VASP)^[^ ^13^ ^]^ were performed to investigate the atomistic mechanism governing the influence of the BSA molecules on the wear of the tribopairs. Given that the BSA molecule, a spheroid protein,^[^ ^14^ ^]^ has a molecular weight of ≈66.4 kDa and is comprised of 583 amino‐acid residues,^[^ ^15^ ^]^ it is difficult to use AIMD to simulate changes in the complete BSA molecule. Therefore, aspartic acid (an important acid at the active site of the BSA molecule) was selected to supplant the BSA molecule as the object of the AIMD simulation to reduce the simulation workload. Based on the experimental results, it can be inferred that Cu doping is the main factor influencing the wear resistance of the TiN*_X_*–Cu films in BSA solutions. Therefore, in this study, the reaction of BSA/TiN*_X_*–Cu was simulated using BSA/Cu.

**Figure 7:**
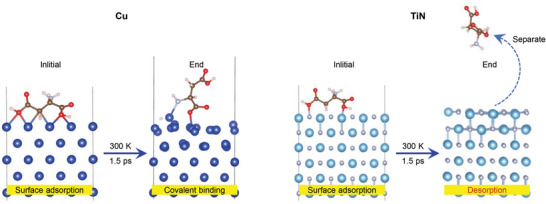
Adsorption of aspartic acid on the surface of Cu and TiN.

The current study focused on the adsorption of aspartic acid on the surfaces of Cu and TiN, and the results are shown in **Figure** **7**. For both models of aspartic acid adsorption, the initial temperature was set to 300 K, and the distance between the acid and surface was identical. Dehydrogenation between aspartic acid and the Cu surface occurred after 1.5 ps of simulation, followed by the formation of metal─carbon bonds. By contrast, under the same conditions, aspartic acid desorbed from the TiN surface, implying that aspartic acid was more stable when adsorbed onto the Cu (TiN*_X_*–Cu) surface than the TiN surface.

**Figure 8:**
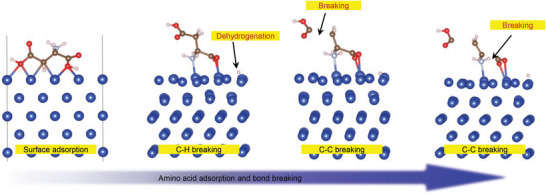
Bond cleavage of aspartic acid on the surface of Cu, as deduced from AIMD simulations.

Starting from an initial configuration consisting of the stable adsorption of aspartic acid on copper, AIMD simulations were conducted using VASP to understand the change in the aspartic acid adsorbed on the Cu surface under the shear force between the tribopairs. The simulation was conducted at 1000 K, which is representative of asperity‐level flash temperatures encountered in tribology experiments.^[^ ^12^ ^]^ As shown in **Figure** **8**, the simulation results indicate that aspartic acid bonded to copper easily dissociates into carbonaceous debris under shear stress. The O─H bond in aspartic acid at the copper surface breaks, and then a stable Cu─O bond forms between the acid and metal (Equation (1)). Afterward, shorter hydrocarbon fragments are formed under a shear force (Equation (2)).

(1) COOH+Cu⇒COO·Cu+H

(2) $$C_{4}H_{6}{\mathrm{NO}}_{4}\Rightarrow \mathrm{COOH}+\mathrm{COO}+C_{2}H_{5}N$$

Erdemir et al.^[^ ^12^ ^]^ proposed that during friction between the MoN*_X_* and Cu coatings in poly‐alpha‐olefin, the linear olefin chains dissociate to form short hydrocarbon fractions, which recombine to form a graphitic tribofilm to lubricate the tribopairs. An important step in the development of graphitic tribofilms from linear olefin chains is the fragmentation of the chains into fractions, followed by their stable absorption onto the surfaces of the tribopairs.

**Figure 9:**
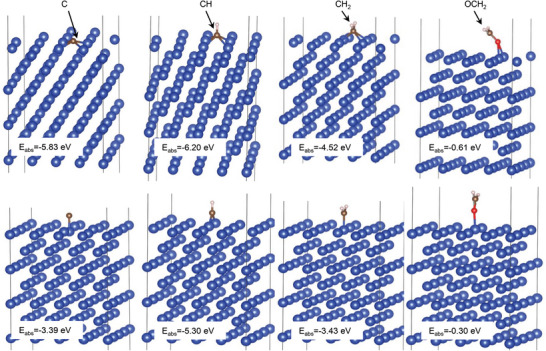
Adsorption energy of different hydrocarbon fragments on the surface of Cu.

The adsorption energies (*E*_abs_) of the different hydrocarbon fragments on the Cu surface were determined using DFT. As shown in **Figure** **9**, the DFT results reveal that the relative position of the fragment on the surface influences its adsorption energy. The *E*_abs_ of C above and over the Cu atom are −5.83 and −3.39 eV, respectively; the *E*_abs_ of CH above and over the Cu atom are −6.20 and −5.30 eV, respectively; and the *E*_abs_ of CH_2_ above and over the Cu atom are −4.52 and −3.43 eV, respectively. The adsorption energies of the C, CH, and CH_2_ fragments on Cu at different positions are less than zero, suggesting that the hydrocarbon fragments were stably adsorbed onto the Cu surface. These hydrocarbon fragments can be utilized as carbon sources to develop graphite‐like carbon films on the tribointerface of the Al_2_O_3_/TiN*_X_*–Cu tribopair. The *E*_abs_ values of various hydrocarbon fragments on the surface of TiN were also determined and are shown in Figure S6, Supporting Information. The hydrocarbon fragments on the TiN surface had a weaker binding strength than those on the Cu surface.

## 3 Discussion

When a metallic hip joint is installed in the human body, a tribofilm is generated at the tribointerface owing to the synergistic effect of friction and catalysis of transition metal ions (such as Co, Cr, and Mo).^[^ ^16^ ^]^ These results indicate that the faster the formation of the tribofilm, the lower the friction and the greater the wear resistance. Therefore, promoting the synthesis of graphite‐like carbon films on the tribointerface to reduce the wear of metal tribopairs is a novel method for extending the lifespan of prostheses. As demonstrated in our previous research, using pure Cu to catalyze the formation of a graphite‐like carbon layer was superior to using a CoCrMo alloy catalyst.^[^ ^17^ ^]^ However, pure Cu is not suitable for use in metallic prostheses.^[^ ^18^ ^]^ In this study, an autoantifriction film (TiN*_X_*–Cu) was prepared using reactive magnetron sputtering to protect the metal substrate from corrosion by bodily fluids and to drive the rapid generation of graphite‐like carbon films. The HRTEM images and X‐ray diffraction (XRD; Figure S7, Supporting Information) patterns show that the deposited autoantifriction film is a single nanocrystalline film containing Cu crystals less than 10 nm in size. Zhang^[^ ^19^ ^]^ showed that doping Cu into a TiN film can reduce the grain size of the material. According to Figure S10, Supporting Information, the TiN film exhibited a columnar structure. However, the TiN*_X_*–Cu films were dense and did not possess columnar structures (Figure S10, Supporting Information). This is due to the refinement of the grains, and the formation of columnar structures in the films can be inhibited by doping with Cu.

Berman et al.^[^ ^20^ ^]^ hypothesized that the tribofilm lubricates the tribopairs and significantly affects their wear behavior. In the current study, the wear resistance of the films was evaluated in BSA solution, and Figure 3 shows that the autoantifriction film had a shallower wear track depth than the TiN film. Therefore, we conclude that the tribofilm on the TiN*_X_*–Cu film is more easily formed than that on the TiN film. Conversely, the Al_2_O_3_/TiN*_X_*–Cu tribopair in BSA solution exhibits a short running‐in period with an elevated and unstable CoF. This is the result of the destruction of microasperities on the contact surface of the Al_2_O_3_/TiN*_X_*–Cu tribopair. Relevant research has demonstrated similar findings.^[^ ^21^ ^]^ Subsequently, CoF reduction results from the following processes. The removal of microasperities polishes the tribointerface of the tribopair, while Cu ions are released from the TiN*_X_*–Cu film; this is followed by the covalent binding of proteins to the surface of the tribointerface, forming a protein layer.^[^ ^17^, ^22^ ^]^ Finally, the combined effects of Cu catalysis and shear forces between the Al_2_O_3_/TiN*_X_*–Cu tribopairs convert the proteins into a graphite‐like carbon film to lubricate the tribopair. The graphite‐like carbon film gradually wears away owing to friction. The CoF of the Al_2_O_3_/TiN*_X_*–Cu tribopair stabilized when the formation and removal of the graphite‐like carbon film reached dynamic equilibrium. The wear mechanism is illustrated in Figure S11, Supporting Information. The formation of a graphite‐like carbon film on the tribointerface is a key factor in enhancing the wear resistance of the TiN*_X_*–Cu films. The wear index is one of the wear‐related material parameters.^[^ ^23^ ^]^ Wang et al.^[^ ^24^ ^]^ deposited Ti─Cu─N coatings on stainless steel using the technique of plasma surface alloying. They prepared Ti─Cu─N coatings with a wear index of ≈3 × 10^−5^ mm^3^ Nm^−1^. The wear index of the TiN*_X_*–Cu film generated in this study was ≈1 × 10^−5^ mm^3^ Nm^−1^ (Figure S3, Supporting Information); therefore, the wear index is superior to that of films developed in previous studies. Because it was difficult to obtain useful information about the tribofilm using optical microscopy, we used ToF‐SIMS to confirm the presence of the tribofilm on the tribointerface. The ToF‐SIMS results (Figure 4) show that the wear scar of the Al_2_O_3_/TiN*_X_*–Cu tribopair can automatically generate a tribofilm composed of carbon and hydrocarbons, which is similar to the findings of Erdemir.^[^ ^12^ ^]^ The structure of the tribofilm on the tribointerface of the Al_2_O_3_/TiN*_X_*–Cu tribopair was characterized using Raman spectroscopy and EELS. Together, the Raman (Figure 5b) and EELS (Figure 6) results suggest that the tribofilm was composed of a carbon film with a structure similar to that of graphite. This graphite‐like carbon film reduced the CoF of the Al_2_O_3_/TiN*_X_*–Cu tribopair (Figure 3d).

Our earlier work^[^ ^17^ ^]^ demonstrated that BSA molecules can be adsorbed onto the Cu surface and denatured into smaller fragments owing to friction between the Al_2_O_3_/Cu tribopairs. DFT and AIMD simulations using VASP were employed to better understand the atomistic mechanism of the influence of BSA molecules on the wear of the tribopairs. As shown in Figures 7 and 8, aspartic acid can covalently bind to the Cu surface and disintegrate into hydrocarbon fragments under shear force. These hydrocarbon fragments can be used as carbon sources to generate graphite‐like carbon films on the tribointerface of a tribopair.

## 4 Conclusion

In this study, we proposed the autoantifriction function of materials placed in biological environments. Using magnetron sputtering, a Cu‐doped TiN film with autoantifriction capability was successfully developed. By coupling the catalysis of copper and shear forces between the tribopairs, the prepared TiN*_X_*–Cu film could independently denature and catalyze protein molecules, resulting in the formation of a graphite‐like carbon tribofilm with anti‐friction and self‐lubrication properties. The developed TiN*_X_*–Cu film demonstrated better wear resistance in protein solutions than the TiN film. Therefore, the autoantifriction TiN*_X_*–Cu film is a promising candidate for modifying the surfaces of metallic artificial hip joints and extending their durability, thereby facilitating the use of catalytic metal‐doped coatings in real‐life medical devices. In vivo environments are substantially more complex than in vitro environments. In our future research, we will conduct animal tests to confirm the autoantifriction function of the TiN*_X_*–Cu film in vivo.

## 5 Experimental Section

### Materials

TiN and TiN*_X_*–Cu films were deposited on polished CoCrMo alloys and Si wafers (Si, 100). To prepare the TiN film, a Ti target (99.95%, 170 × 135 × 6 mm^3^) was sputtered using direct‐current magnetron sputtering. The TiN*_X_*–Cu film was obtained by sputtering a Cu─Ti target containing 32 Cu rods (99.95%, *Φ* = 5 mm, *H* = 6 mm) in a mosaic configuration on the Ti target racetrack. Before depositing the films, the substrates were cleaned using Ar plasma for 30 min to activate and clean the surfaces of the CoCrMo alloy and Si. To improve the adhesion between the films and substrates, a Ti interlayer with a thickness of ≈200 nm was deposited on the substrates. Ar (99.999%, 40 sccm) and N_2_ (99.999%, 6 sccm) were used as working gases. The TiN and TiN*_X_*–Cu films were deposited at a DC bias voltage of −50 V for 30 min. Throughout the film‐deposition process, the vacuum‐chamber pressure, substrate‐to‐target distance, and target sputtering power were maintained at ≈0.5 Pa, 80 mm, and 500 W, respectively.

### Characterization

The cross‐sectional image of the prepared TiN*_X_*–Cu film was acquired using a FIB (FEI STRATA 400S). The crystalline structures of the films were characterized using XRD (PANalytical, X'pert) with Cu‐K_α_ radiation generated at 40 kV and 20 mA from a Cu target in *θ*–2*θ* mode. The microstructures of the TiN*_X_*–Cu films were investigated using TEM (FEI Titan G2 60‐300). ToF‐SIMS (ION TOF‐SIMS 5) was used to investigate the composition of the tribofilm formed on the wear scars. The surface morphologies of the films were investigated using scanning electron microscopy (SEM, JSM‐7001F, JEOL, Japan). The Cu content of the TiN*_X_*–Cu films was analyzed using EDS. The surface roughness of the films and Al_2_O_3_ balls was measured using atomic force microscopy (Multimode 8, Bruker, USA), and the *R*_q_ (root mean square) was used to quantify the surface roughness. Raman microscopy (*λ* = 532 nm, inVia Raman Microscope, RENISHAW) and EELS (JEOL 2100F) were used to characterize the structure of the tribofilm formed on the wear interface of the tribopairs.

### Wear Testing

As a representative wear‐testing method, ball‐on‐disk wear testing was widely used to evaluate the wear resistance of bearing couples in artificial joints.^[^ ^25^ ^]^ In this study, friction and wear tests were performed on the TiN and TiN*_X_*–Cu films using a ball‐on‐disk reciprocating tribometer (CSEM, Switzerland) in saline (NaCl, 9 mg mL^−1^) and BSA solutions (composition: BSA, 20 mg mL^−1^, EDTA, 2 mg mL^−1^, sorbic acid, 0.2 mg mL^−1^, and NaCl, 9 mg mL^−1^) at a room temperature of ≈25 °C. Tribopairs were formed by pressing Al_2_O_3_ balls (*Φ* = 6 mm) into TiN‐ or TiN*_X_*–Cu‐film‐coated CoCrMo alloy discs under a load of 0.5 N for 100 000 cycles in BSA solution and 10 000 cycles in saline solution. The sliding frequency was 1 Hz, and the length of the wear track was ≈6 mm. Using the ball‐on‐plate geometry (Figure S8, Supporting Information), the Hertzian contact theory was used to determine the maximum contact pressure (*σ*_c_)_max_ between the Al_2_O_3_ balls and film‐coated CoCrMo alloy discs. In this study, (*σ*_c_)_max_ at the beginning of wear testing was estimated using Equations (3)–(5).^[^ ^26^ ^]^

(3) $$\frac{1}{E^{*}}=\frac{1}{2}\left(\frac{1-\nu_{1}^{2}}{E_{1}}+\frac{1-\nu_{2}^{2}}{E_{2}}\right)$$

(4) $$a≅{\left(\frac{3RF}{2E^{*}}\right)}^{1/3}$$

(5) $${\left(\sigma_{c}\right)}_{\max}≅\frac{3F}{2\pi a^{2}}$$

where *ν*_1_ and *ν*_2_ were the Poisson's ratios, *E*_1_ and *E*_2_ were the elastic moduli associated with the tribopairs, E^*^ was the reduced elastic modulus (Equation (1)), *a* was the depth of indentation at the start of wear testing, *R* was the radius of the Al_2_O_3_ balls used in this study, and *F* was the load exerted on the tribopairs. The properties of the Al_2_O_3_ ball and CoCrMo alloy provided by the manufacturers are listed in Table S1, Supporting Information. The contact pressure between the Al_2_O_3_ ball and the CoCrMo alloy disk was recorded at 337.1 MPa. The wear index *W*_V_ of the films was calculated using Equation (6).^[^ ^27^ ^]^

(6) $$W_{V}=\frac{V}{F\cdot s}$$

where *V* was the volume of the removed material in cubic millimeters (mm^3^), *F* was the load exerted on the tribopairs, and *s* was the sliding distance in meters. All quantitative data obtained in this study were analyzed using one‐way ANOVA and were expressed as mean values ± standard deviations.

### Calculation

In this study, DFT and AIMD calculations were conducted using VASP,^[^ ^13^ ^]^ in which the projector augmented‐wave method was implemented. The Perdew (Burke) Ernzerhof functional was adopted as the exchange‐correlation functional.^[^ ^28^ ^]^ An energy cut‐off of 450 eV was used for the plane‐wave basis set. The energy and force convergence criteria were set to 10^−6^ eV and 0.01 eV Å^−1^, respectively. A gamma‐only k‐mesh was used in the calculations to sample the Brillouin zone of a slab adsorbed with a molecule. Moreover, to avoid possible interactions between the repeated slabs induced by periodicity, a 15–Å vacuum was added in the direction perpendicular to the surface.

## Conflict of Interest

The authors declare no conflict of interest.

## Author Contributions

Q.Y.D. and Q.G.F. contributed equally to this work. Y.X.L. conceived and designed this study. Q.Y.D. and M.T.L. wrote the original manuscript. Q.Y.D., D.L.M., Y.T.L., P.P.J., and Y.L.G. performed the experiments, and Q.G.F. performed the calculation. M.T.L., F.W., and Y.X.L. reviewed the manuscript. All the authors discussed the data reported in the paper.

## Supporting information

Supporting Information

## Data Availability

The data that support the findings of this study are available from the corresponding author upon reasonable request.
