# Supplementary material for: Metal‐Driven Autoantifriction Function of Artificial Hip Joint
Source: Adv Sci (Weinh). 2023 Jul 6;10(25):2301095. doi: 10.1002/advs.202301095 (PMC10477871; doi:10.1002/advs.202301095)
Supplement: Supplementary file 1 — Supporting Information [file ADVS-10-2301095-s001.pdf]

ADVANCED SCIENCE
Open Access

Supporting Information

for *Adv. Sci.*, DOI 10.1002/advs.202301095

Metal-Driven Autoantifriction Function of Artificial Hip Joint

Qiaoyuan Deng, Qingguo Feng, Peipei Jing, Donglin Ma, Mengting Li, Yanli Gong, Yantao Li, Feng Wen and Yongxiang Leng**

Supplemental Material for

Metal-driven autoantifriction function of artificial hip joint

Q.Y. Deng[1, 2], Q.G. Feng [1], P.P. Jing[1], D.L. Ma[3], M.T. Li[4*], Y.L. Gong[1], Y.T. Li[1], F. Wen[2], Y.X. Leng[1*]

1. Institute of Biomedical Engineering, Key Laboratory of Advanced Technologies of Materials, Ministry of Education, College of Medicine, Southwest Jiaotong University, Chengdu 610031, Sichuan, China

2. Key Laboratory of Advanced Material of Tropical Island Resources of Educational Ministry, School of Materials Science and Engineering, Hainan University, Haikou, Hainan 570228, China

3. College of Physics and Engineering, Chengdu Normal University, Chengdu, Sichuan 611130, China

4. Hainan Provincial Fine Chemical Engineering Research Center, Hainan University, Haikou, Hainan 570228, PR China

*Corresponding authors:

Prof. Dr. Y.X. Leng, Email: yxleng@263.net

Dr. M.T. Li, Email: limengting@hainanu.edu.cn

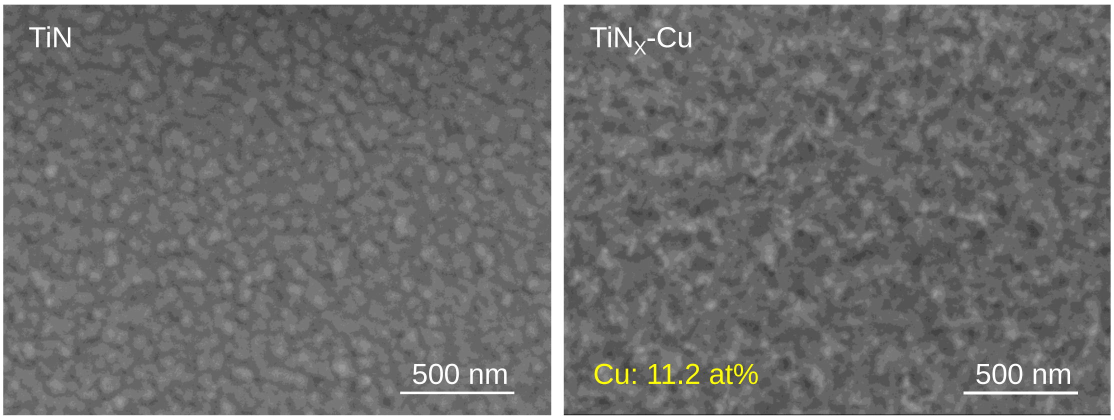

Supplementary Figure 1 SEM images of the TiN and TiN_X-Cu films

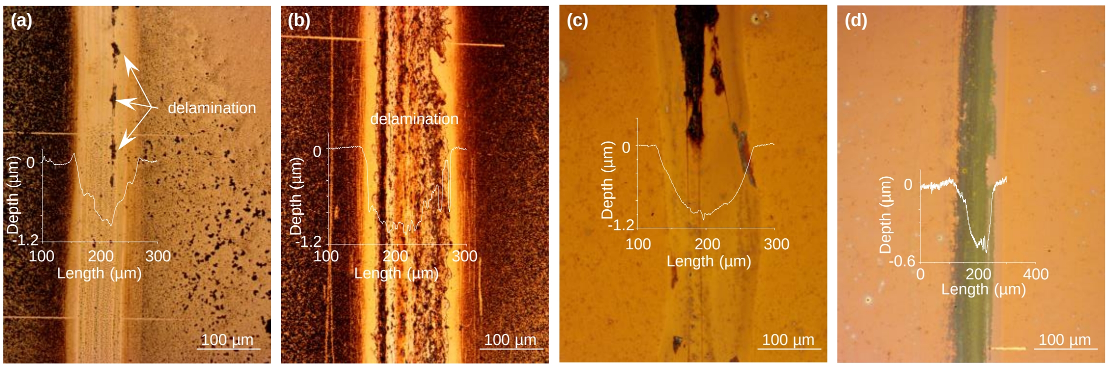

Supplementary Figure 2 Optical image and the profile of the films. (a) TiN film in saline; **(b)** TiN_X-Cu film in saline; **(c)** TiN film in BSA solution; **(d)** TiN_X-Cu film in BSA solution.

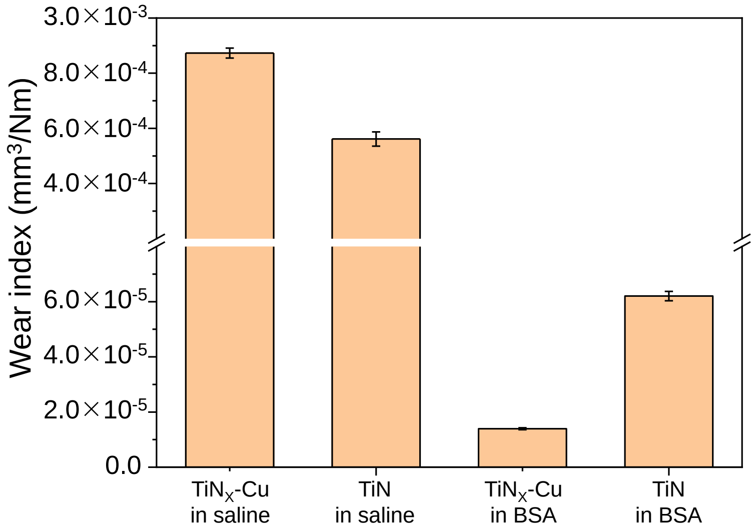

Supplementary Figure 3 Wear index of the TiN and TiN_X-Cu films in saline (10, 000 cycles) and in BSA solution (100, 000 cycles)

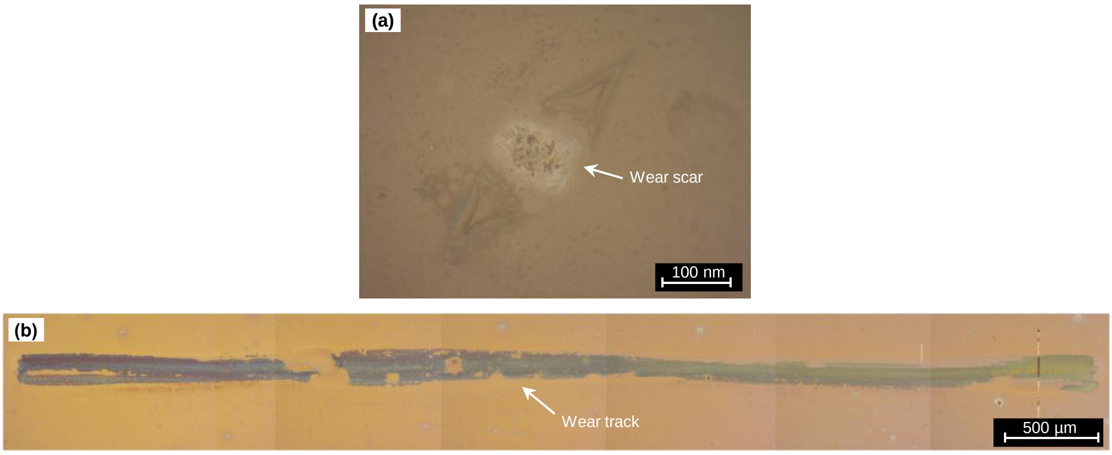

Supplementary Figure 4 Optical images of the Al_2O_3/TiN_X-Cu tribo-pairs. (a) The wear scar formed on the Al_2O_3 ball after the wear testing in BSA solution. **(b)** The wear track formed on the TiN_X-Cu film after the wear testing in BSA solution.

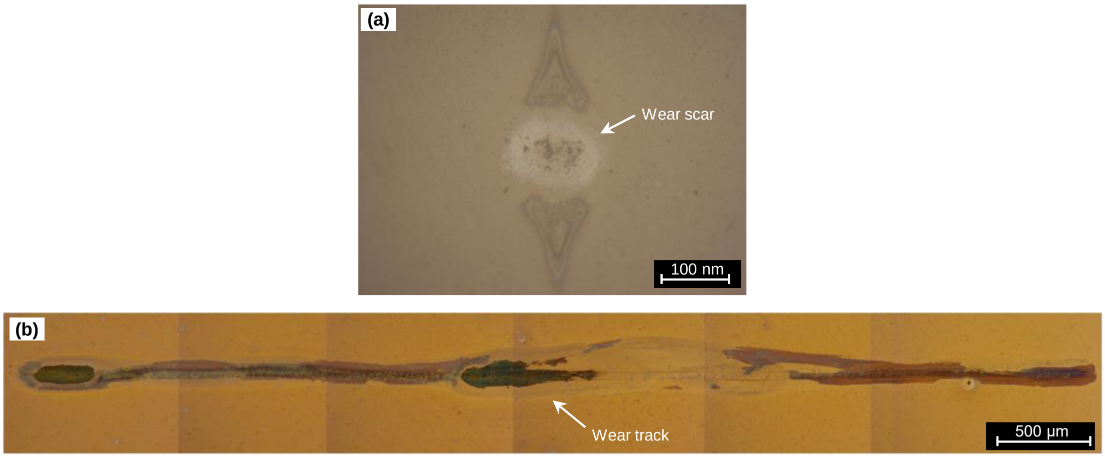

Supplementary Figure 5 Optical images of the Al_2O_3/TiN tribo-pairs. (a) The wear scar formed on the Al_2O_3 ball after the wear testing in BSA solution. **(b)** The wear track formed on the TiN film after the wear testing in BSA solution.

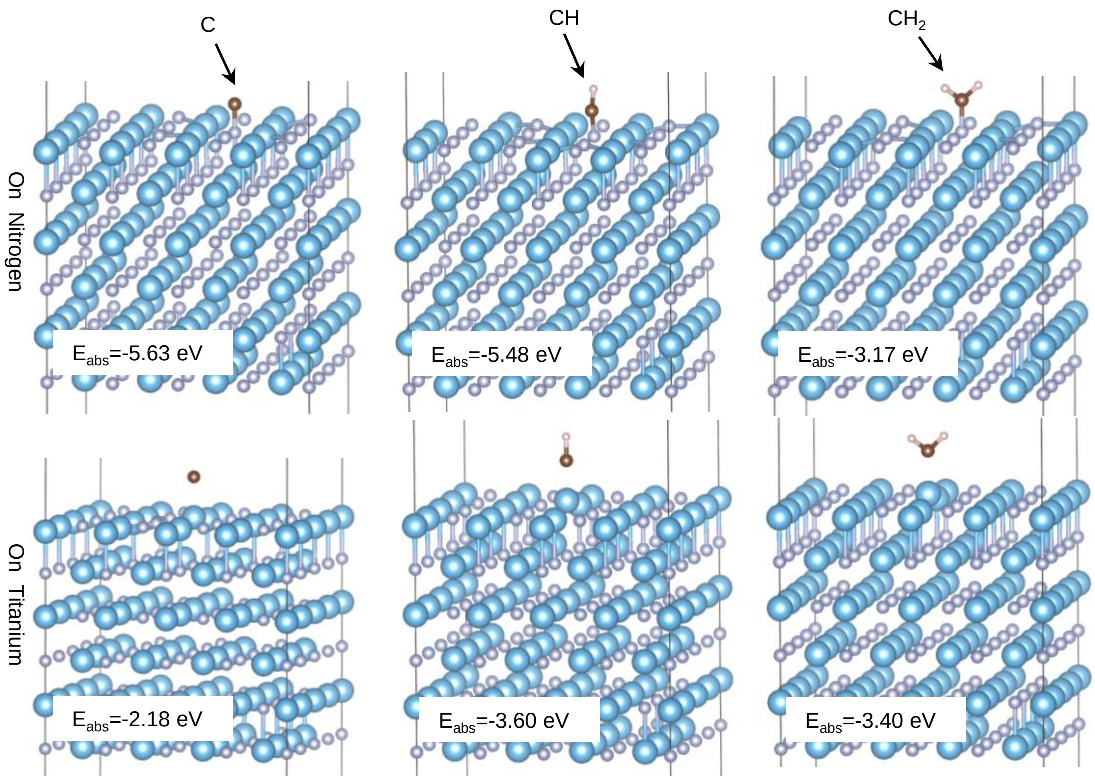

Supplementary Figure 6 Adsorption energy of various hydrocarbon fragments on the surface of TiN.

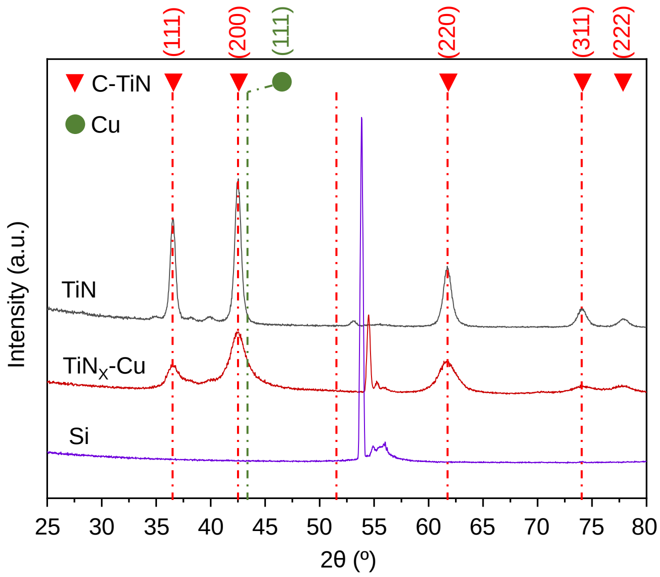

Supplementary Figure 7 X-ray diffraction characterization of Si substrate, TiN_X-Cu film and TiN film.

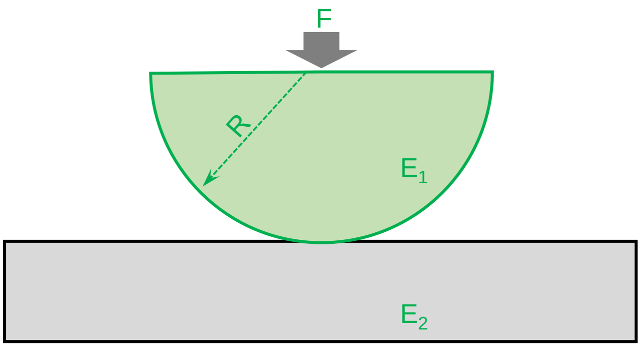

Supplementary Figure 8 Theoretical model to estimate the contact pressure of the tribo-pairs.

Supplementary Table 1. The properties of materials used for wear testing. Parameters from the manufacturers.

Material	Young modulus (GPa)	Poisson's ratio
Al_2O_3 ball	300	0.20
CoCrMo alloy	177	0.29

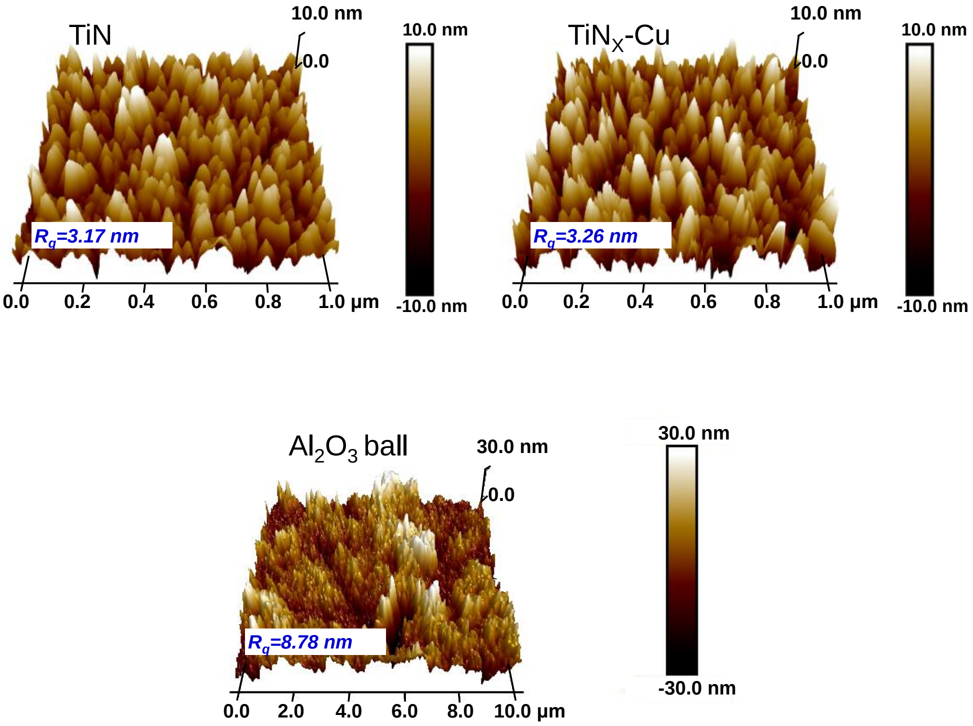

Supplementary Figure 9 Surface morphology of TiN, TiN$_X$-Cu films and Al_2O_3 ball

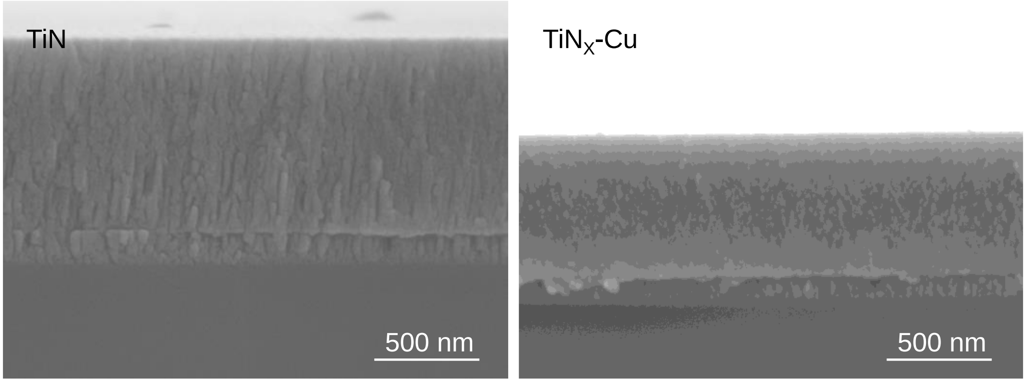

Supplementary Figure 10 Cross section view of of the TiN and TiN_X-Cu films

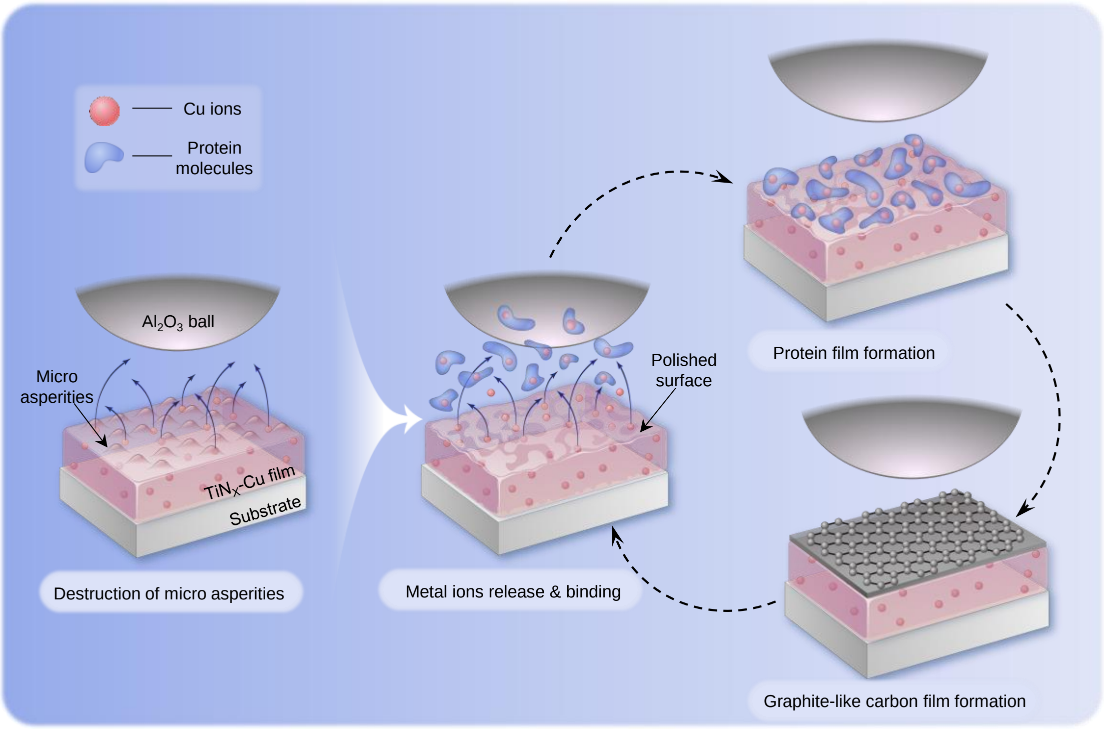

Supplementary Figure 11 Illustration of the wear mechanism of TiN_X-Cu films in BSA solution
